# Enhanced cell dehydration tolerance and photosystem stability facilitate the occupation of cold alpine habitats by a homoploid hybrid species, *Picea purpurea*

**DOI:** 10.1093/aobpla/ply053

**Published:** 2018-09-07

**Authors:** Jingru Wang, Minghao Wang, Xiaowei Zhang, Shan Sun, Aiping Zhang, Ning Chen, Changming Zhao

**Affiliations:** 1 State Key Laboratory of Grassland Agro-Ecosystems, School of Life Sciences, Lanzhou University, Lanzhou, China; 2 Forestry College of Gansu Agricultural University, Lanzhou, China

**Keywords:** Cell dehydration, frost tolerance, homoploid hybrid speciation, maximum photochemical efficiency of photosystem II, *Picea purpurea*, water relations, xylem resistance to embolism

## Abstract

Homoploid hybrid speciation (HHS), characterized by hybrid speciation without a change in chromosome number and facilitated by ecological divergence, is well known in angiosperms but rare in gymnosperms. *Picea purpurea* as one of two demonstrably conifer diploid hybrid species in gymnosperms has been found to occupy colder alpine habitats than its parents. However, studies on whether leaf frost tolerance and hydraulic safety exhibit transgressive segregation and thus play a role in conifer HHS are still lacking. In this study, we compared the frost tolerance of photosystem stability (the maximum efficiency of PSII, *F*_v_/*F*_m_), pressure-volume parameters, and xylem resistance to dysfunction of leaves (current-year twigs) and stems (annual shoots) between *P*. *purpurea* and its progenitors. The results indicated that *P. purpurea* had significantly lower osmotic potential at full turgor, water potential at turgor loss point, water potential at 12 % loss of conductance of stem, the maximum hydraulic conductance of stem and the temperature causing a 50 % reduction in initial *F*_v_/*F*_m_ than its parental species. In contrast, the leaf and stem xylem pressure inducing 50 % loss of hydraulic conductivity (leaf Ψ_50_ and stem Ψ_50_, respectively) and hydraulic safety margin in leaf Ψ_50_, stem Ψ_50_ in *P. purpurea* showed no significant difference with those of *P. wilsonii*, but significantly larger than those of *P. likiangensis*. This suggests that the frost tolerance of photosystem stability and the cell dehydration tolerance in *P. purpurea* are superior to its parental species, facilitating its successful colonization and establishment in colder habitats.

## Teaser

It has been shown that *Picea purpurea* originated from hybrid speciation with no change in chromosome number, and occupies higher altitudes and latitudes than its parental species. However, a few cases studied the transgressive segregation in frost tolerance and resistance to cold-induced drought, in terms of physiological traits (e.g. water relations and xylem vulnerability to embolism) between the hybrid and its parental species. Our results revealed that *P. purpurea* exhibited greater cell dehydration tolerance and frost tolerance of photosystem stability than its parental species, suggesting that these characteristics facilitated its successful establishment in alpine regions.

## Introduction

Homoploid hybrid speciation (HHS) entails the establishment of novel lineages through hybrid speciation with no change in chromosome number, and is facilitated by ecological divergence. It is well known in angiosperms but rare in gymnosperms (Mallet 2007; Yakimowski and Rieseberg 2014). To date, only two conifer species, *Pinus densata* and *Picea purpurea*, are known to have originated from this pattern of speciation, and both of them occupy colder alpine habitats than their parental species (Wang *et al.* 2011; Sun *et al.* 2014). Homoploid hybrid speciation has primarily been explained in terms of transgressive segregation (Rieseberg *et al.* 1999, 2007; Lexer *et al.* 2003; Gross and Rieseberg 2005). For instance, seedlings of *P. densata* exhibit greater total dry mass production and long-term water use efficiency under drought stress than those of its progenitors, which facilitated its successful colonization and establishment in high-altitude regions where the cold-induced drought stress may affect the growth of plants (Ma *et al.* 2010). However, species distributions along altitude gradients are potentially determined by differential frost tolerance and resistance to cold-induced drought conferred by physiological or morphological traits such as increased cell survival at low water potentials and reduced vulnerability of xylem vessels to embolism (Baltzer *et al.* 2005). This is particularly important for homoploid hybrid species: the transgressive expression of such traits enables the initial hybrid generation to occupy novel habitats and establish niche separation from its progenitors, thereby avoiding the homogenizing effects of gene flow and competition from the parental species (Rieseberg *et al.* 2003; Gompert *et al.* 2006). To date, quantitative studies on transgressive segregation in the frost tolerance and drought tolerance of conifers in homoploid hybrid species, and its potential contribution to their establishment in cold habitats, are lacking.

The frost resistance of needles depends on the protoplast’s tolerance of dehydration resulting from extracellular ice formation during freezing stress (Beck *et al.* 2007). Plants usually improve their cellular dehydration tolerance by osmotic adjustment, i.e. by raising their intracellular solute concentrations to reduce the cells’ osmotic potential at full turgor (*π*_o_) (Patakas and Noitsakis 1997). Accordingly, the review by Bartlett *et al.* (2012) and a more recent research paper by Maréchaux *et al.* (2015) both suggested that the leaf water potential at cell turgor loss (Ψ_TLP_) could be used to quantify cellular dehydration tolerance in plants of a given species; several studies have shown that *π*_o_ and Ψ_TLP_ are strongly correlated (Sack *et al.* 2003; Lenz *et al.* 2006; Scoffoni *et al.* 2011; Bartlett *et al.* 2012; Maréchaux *et al.* 2015). Species with low Ψ_TLP_ values tend to have larger ranges of water potentials at which the leaves remain turgid and retain functions such as stomatal conductance, photosynthetic gas exchange and hydraulic conductance (Sack *et al.* 2003; Baltzer *et al.* 2008; Blackman *et al.* 2009, 2010).

Freezing-induced water stress can also reduce water transport to the canopy as a result of xylem embolism formation due to the high solar radiation increasing the needles’ transpiration demands and frozen soil and stem blocking the water uptake during winter (Pearce 2001; Mayr *et al.* 2006; Beck *et al.* 2007). The xylem’s vulnerability to embolism is often evaluated by plotting a hydraulic vulnerability curve, which describes the relationship between the xylem pressure and percentage losses of conductance (Sperry and Pockman 1993). The xylem pressures inducing 12, 50 and 88 % losses of hydraulic conductance (Ψ_12_, Ψ_50_ and Ψ_88_, respectively) can be obtained from this curve, and used to compare xylem vulnerability across species, within different organs of one species, and within species across environmental gradients (Sperry and Pockman 1993; Pockman and Sperry 1997; Tyree and Sperry 1988). Ψ_12_ has been described as the upper vulnerability threshold and is related to the stomatal control of transpiration, which ensures that the maximum xylem tension does not exceed this threshold value for the stem (to which the leaves are attached) under non-extreme conditions (Sparks and Black 1999; Brodribb and Holbrook 2003; Domec *et al.* 2008; Delzon and Cochard 2014). In contrast, Ψ_50_ and Ψ_88_ are often used as indicators of resistance to catastrophic xylem failure under extreme water scarcity (Maherali *et al.* 2004, 2006). Additionally, various hydraulic safety margins are used to describe the degree of conservatism in a plant’s strategies (Meinzer *et al.* 2009; Johnson *et al.* 2012). One such safety margin is the difference between Ψ_50_ and the minimum xylem pressure a stem experiences over a day (often referred to as the xylem pressure at midday, Ψ_mid_), which shows how close a species’ hydraulic operating conditions are to the steepest point of its vulnerability curve, and hence a potentially catastrophic embolism (Johnson *et al.* 2012). Hydraulic safety margins can also be computed for specific organs. For example, the difference between the stem Ψ_50_ and the leaf Ψ_50_ can be related to the magnitude of the difference between the leaf and stem hydraulic safety margins (Johnson *et al.* 2012).

Needles are the sites of photosynthesis in conifers, so if they freeze in winter, the photosystems’ stability will be reduced and there will be a decline in the maximum photochemical efficiency of photosystem II (PSII) (evaluated as the ratio of variable to maximum chlorophyll fluorescence, *F*_v_/*F*_m_) (Robakowski 2005). An apparent decline in *F*_v_/*F*_m_ during freezing stress has been observed in some conifers and many evergreen species (Oliveira and Peñuelas 2004; Robakowski 2005). Compared to species from warm regions, species in cold alpine habitats tend to exhibit greater photosystem stability (i.e. a lower temperature causing a 50 % reduction in *F*_v_/*F*_m_) at a given freezing temperature (Cunningham and Read 2006). It is therefore reasonable to expect a diploid conifer hybrid species inhabiting an extremely cold environment to exhibit greater needle frost tolerance, water relations and hydraulic safety to water stress than its progenitors.

There is molecular evidence that *P. purpurea* originated from the hybridization of *P. wilsonii* and *P. likiangensis* with no change in chromosome number, and it is distributed in northeastern Qinghai-Tibet Plateau (QTP) at higher altitudes and latitudes than its parental species (Sun *et al.* 2014). According to the *Flora of China* and our previous research (Wang *et al.* 2018), *P. purpurea* prefers habitats with cold and humid climates, while its parents prefer habitats with warm or mild climates (Cheng and Fu 1978; Table 1). Consequently, these three species collectively constitute a unique model system for studying the mechanisms associated with segregation in the hydraulic safety parameters and frost tolerance of needles that enable homoploid hybrid species to colonize cold habitats. The water relations and the vulnerability of xylem to embolism of all three species were investigated by examining their pressure-volume curves and vulnerability curves, respectively. In addition, a freezing temperature response experiment was conducted to compare the frost tolerance of needles from *P. purpurea* and its parental species by measuring their photosystem stability (*F*_v_/*F*_m_). We hypothesized that *P. purpurea* would exhibit transgressive segregation from its progenitors with respect to needle frost tolerance, water relations and hydraulic safety to water stress, and that these transgressions facilitated its colonization of colder alpine habitats.

**Table 1.** Comparison of main climate characteristic between *Picea purpurea* and its parental species. Significantly interspecific differences are indicated with different letters (*P* < 0.05). Data are presented as means ± SE.

Environmental variables	*P. purpurea*	*P. wilsonii*	*P. likiangensis*
Annual mean temperature (°C)	3.72 ± 0.32 b	8.64 ± 0.36 a	7.68 ± 0.35 a
Max temperature of warmest month (°C)	18.71 ± 0.34 b	26.4 ± 0.31 a	19.37 ± 0.3 b
Min temperature of coldest month (°C)	−15.55 ± 0.44 c	−10.86 ± 0.59 b	−8.51 ± 0.46 a
Annual precipitation (mm)	736.24 ± 19.35 b	663.71 ± 23.42 c	799.86 ± 17.46 a
Precipitation of wettest month (mm)	135.6 ± 3.31 b	139.24 ± 3.27 b	190.57 ± 4.16 a
Precipitation of driest month (mm)	3.67 ± 0.22 b	6.46 ± 0.5 a	3.07 ± 0.15 c

## Materials and Methods

### Plant material

Seeds of *P. purpurea*, *P. wilsonii* and *P. likiangensis* were collected from their central geographical distributions to obtain samples that reflect the distinct characteristics of their preferred habitats. During this process, 10–15 matured trees were selected at each site, and all trees sampled were at least 100 m apart. These seeds were then germinated and grown in a tree nursery (Yuzhong, Gansu province; 35°56.61′N, 104°09.079′E; 1750 m a.s.l.; mean annual temperature 7.1 °C; mean growing-season temperature, 13 °C) until they reached 5 years of age. Before starting the experiments, 10 promising and uniform seedlings of each species were randomly selected and replanted into pots containing a homogeneous mixture to facilitate the planned measurements (Table 2). All measurements were performed under well-watered conditions to enable reliable evaluation of genetically based differences.

**Table 2.** Descriptive of current-year twigs and seedlings growth performance for *Picea purpurea* and its parental species. Significantly interspecific differences are indicated with different letters (*P* < 0.05). Data are presented as means ± SE.

Species	Twigs diameter (mm^2^)	Twigs length (mm)	Seedlings diameter (mm^2^)	Seedlings height (cm)
*P. purpurea*	1.64 ± 0.10 a	36.73 ± 1.12 c	190.94 ± 13.86 a	32.29 ± 1.77 a
*P. wilsonii*	1.66 ± 0.16 a	53.63 ± 1.32 b	158.41 ± 10.10 a	30.20 ± 1.67 a
*P. likiangensis*	1.59 ± 0.12 a	61.73 ± 3.58 a	160.79 ± 24.75 a	38.00 ± 4.77 a

### Measurement of pressure-volume curves and xylem vulnerability curves

Branches with annual shoots and current-year twigs were collected from 3–5 individuals per species during the growing season in August 2013 and used to gather measurements for the construction of pressure-volume (P-V) and xylem vulnerability curves. Material was collected at night to minimize the risk of significant transpirational water loss. The resins of *Picea* branches can clog the cut surface area, which may lead to erroneous results during water potential and hydraulic measurements. Thus, the collected current-year twigs and branches were recut under water and then the cut surface was plunged into boiling water for 60 s to kill the resin-producing parenchyma cells (Waring and Silvester 1994). After that, the twigs and branches were rehydrated for 8 h and 48 h, respectively, prior to use.

The leaf P-V curves of all three species were constructed using current-year twigs following the bench dehydration method with a pressure chamber (PMS Instrument Co., Corvallis, OR, USA) and fitted as described by Sack *et al.* (2003). The twigs’ dry masses were then determined by drying the samples at 65 °C for 72 h. Six leaf P-V parameters were then calculated for the subsequent analysis (Koide *et al.* 1989): water content at full turgor (SWC), leaf osmotic potential at full turgor (*π*_o_), water potential and relative water content at the turgor loss point (Ψ_TLP_ and RWC_TLP_, respectively), bulk modulus of elasticity (*ε*) and relative capacitance at full turgor (*C*_FT_). In addition, the shoots’ relative water contents (RWC %) were determined from their P-V curves using the expression (*M*_f_*− M*_d_)/(*M*_ws_*− M*_d_) * 100, where *M*_f_ is the fresh mass, *M*_d_ is the dry mass (after >72 h at 65 °C) and *M*_ws_ is the water-saturated mass.

Xylem vulnerability curves for annual shoots were determined by the dehydration method (Sperry *et al.* 1988). Branches were dried for varying lengths of time to generate a range of xylem water potentials. The hydraulic conductance of the stem was measured using a water conductance apparatus comprising a bottle of deionized water (de-gassed in advance under reduced pressure for at least 30 min) fastened to a wall at a height of 1 m above the stem such that gravity causes water to flow into the xylem. A glass pipette (25 °C, 2 mL) was used to calculate the flow rate (*V*/*t*, where *V* = volume, *t* = flow time of volume) at the end of the stem. A short segment ~2.5–3 cm in length was cut from an annual shoot under water (to prevent air entry) and mounted on this apparatus to measure its hydraulic conductance before and after high-pressure embolism removal (*K* and *K*_max_, respectively). The water potential of each segment was determined in current-year twigs within one branch. Before each measurement, the branch was wrapped in a black plastic bag to equilibrate the water potential of the xylem for at least 30 min. The percent loss of conductivity (PLC %) was then calculated using the following equation:

$$PLC\;\%=\text{ 1}00\times (K_{\text{max}}-K)/K_{\text{max}}$$ (1)

The vulnerability curve was then obtained using the following sigmoid function (Pammenter and Vander Willigen 1998):

$$PLC\;\%=\text{1}00/(\text{1}+{\text{e}}^{a}{{}^{*}}^{\left(\Psi -b\right)})$$ (2)

where *a* is the curve’s maximum slope, *b* is the water potential of the xylem at 50 % loss of conductance and Ψ is the xylem water potential before and after embolism removal (Pammenter and Vander Willigen 1998). In addition, the midday water potential (Ψ_mid_) of each species was obtained from current-year twigs in well-watered seedlings using a pressure chamber on a cloudless day.

Measurements of leaf hydraulic conductance (*K*_leaf_) were performed using the evaporative flux method of Sack *et al.* (2003) with current-year twigs from the same branch that had been used for P-V curve determination. Flow rates were measured using a PC-connected flow meter (μ-Flow 0.2 g h^−1^, Bronkhorst High Tech, The Netherlands), logging data every 30 s. For each twig, recording was allowed to proceed until the coefficient of variation remained <5 % for 10 min (indicating the onset of a steady state). The twigs’ masses were weighed before and after the flow rate measurements. Twigs were then dried on a bench and bagged for 0.5 h to equilibrate before the next measurement. The *K*_leaf_ was calculated as:

$${Ｋ}_{\text{leaf}}＝F/(A*(0–P_{\text{leaf}-\text{fin}}))$$ (3)

where *F* is the flow rate, *A* is the leaf area of the branchlet and *P*_leaf-fin_ is the leaf water potential after rehydration.

Because the twigs used for *K*_leaf_ measurements had experienced a short rehydration (~10 min), which would affect their later water potential measurements, the relationship between the water potential and *K*_leaf_ was characterized based on the measured RWC % values and the water potentials obtained from the P-V curves. For each *K*_leaf_, the lowest water potential was selected. Then, the vulnerability curves were obtained by plotting PLC against the water potential, where the PLC was calculated after defining the maximum hydraulic conductance for each species based on the relationship between hydraulic conductance and water potential.

### Chlorophyll fluorescence measurements

The needles’ frost tolerance was estimated by measuring their fluorescence in winter. Detached needles of five plants per species taken from current-year twigs were sealed in a zip lock bag and exposed to the temperature of −40, −30, −20, 5, 10, 15, 20 and 25 °C for at least 30 min, respectively. During the treatment, needles were kept in a dark room to facilitate the fluorescence measurement. The photochemical efficiency of PSII (*F*_v_/*F*_m_) was then measured through a portable pulse amplitude modulated (PAM) fluorimeter (FMS-2, Hansatech, UK). The response curves of *F*_v_/*F*_m_ to temperature were plotted and then fitted using a Gompertz equation (Equation 4):

$$y=a*e^{e^{-(T-c)/b}}$$ (4)

where *y* is the percentage reduction in *F*_v_/*F*_m_ relative to the initial value, *T* is the treatment temperature, and *a*, *b* and *c* are fitting parameters. The regression was then used to estimate the temperatures that reduced the *F*_v_/*F*_m_ for each species to 50 % of its initial value (FT_50_; Cunningham and Read 2006).

### Statistical analysis

The statistical significance of inter-species differences in the studied variables was evaluated by one-way analysis of variance (ANOVA) using a least significance difference (LSD) test, with a significance threshold of *P* < 0.05 (SPSS 16.0; SPSS Inc.). The homogeneity of the variances was verified before performing ANOVA. Curves were fitted and plotted using SigmaPlot 12.5 (Systat Software, Inc.).

## Results

### Variation in pressure-volume traits

Significant differences between *P. purpurea* and its parental species were observed for three of the six leaf P-V parameters (Table 3): the shoot water content at full turgor (SWC), osmotic potential at full turgor (*π*_o_) and water potential at turgor loss point (Ψ_TLP_) of *P. purpurea* were all lower than those of *P. wilsonii* and *P. likiangensis*. Additionally, the value of *π*_o_ for *P. wilsonii* was less negative than that for *P. likiangensis*. No significant inter-species differences were detected in the remaining parameters.

### Variation in xylem vulnerability

**Figure 1.**
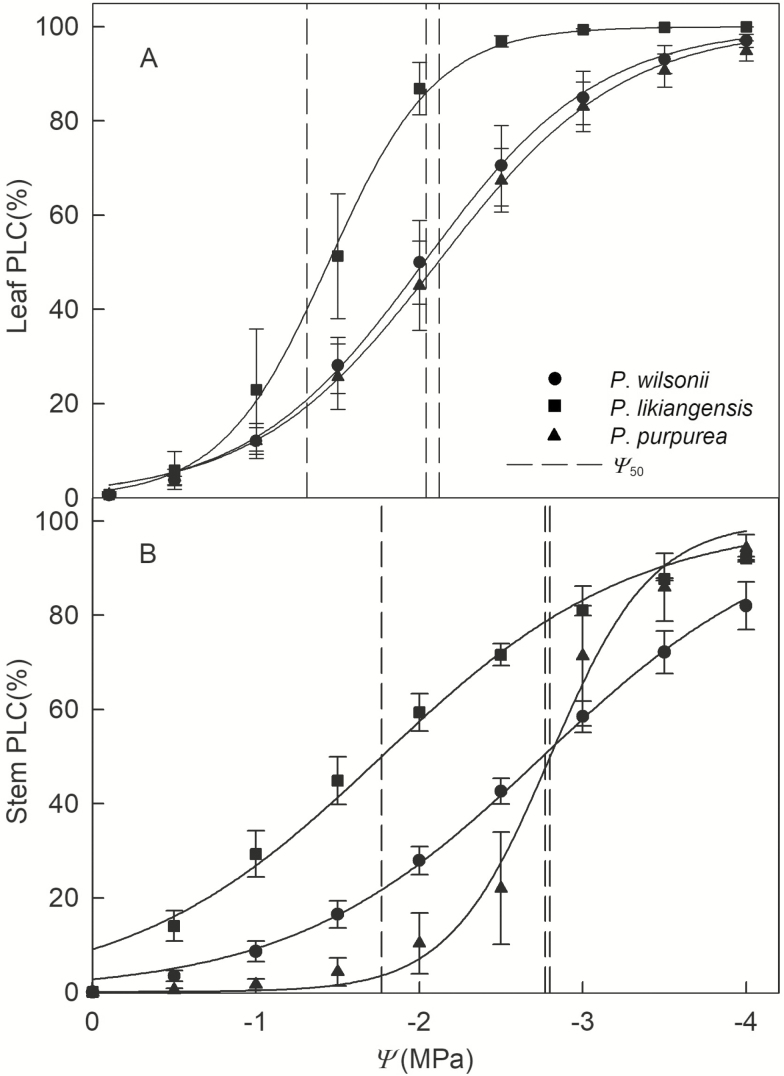
Percent loss of conductance versus water potential (Ψ) for leaves (A) and stems (B) of *Picea purpurea*, *P. wilsonii* and *P. likiangensis* (represented by triangles, circles and squares, respectively). Curves were fit using all data. Data points are presented as means ± SE.

Vulnerability curves for leaves and stems (i.e. current-year twigs and annual shoots, respectively) indicated great variation within each species (Fig. 1A and B). In addition, *P. purpurea* and *P. wilsonii* had more negative Ψ_50_ values for both leaves and stems than *P. likiangensis* (Table 3; Fig. 1A and B). Significant inter-species differences in the xylem pressures inducing 12 % loss of hydraulic conductivity (Ψ_12_) were observed for stems, but not for leaves (Table 3): *P. purpurea* exhibited more negative stem Ψ_12_ values than its progenitors (Table 3). Conversely, there were significant inter-species differences in the xylem pressure inducing 88 % loss of hydraulic conductivity (Ψ_88_) for leaves, but not for stems (Table 3). No significant inter-species differences were detected in Ψ_mid_ or leaf *K*_max_ (Table 3). Nonetheless, *P. purpurea* had the lowest stem maximum hydraulic conductance (stem *K*_max_) of the three species, and the stem *K*_max_ of *P. likiangensis* was three times higher than that of *P. purpurea* (Table 3). The safety margin between the midday water potential and xylem pressure inducing 50 % loss of leaf hydraulic conductance (Ψ_mid_ − leaf Ψ_50_) in *P. purpurea* was slightly larger than in *P. wilsonii* and significantly greater than in *P. likiangensis* (Table 3). However, there were no significant inter-species differences in the difference between leaf Ψ_50_ and stem Ψ_50_ (leaf Ψ_50_ − stem Ψ_50_; Table 3).

### Frost tolerance of PSII photochemical efficiency in leaves

**Figure 2.**
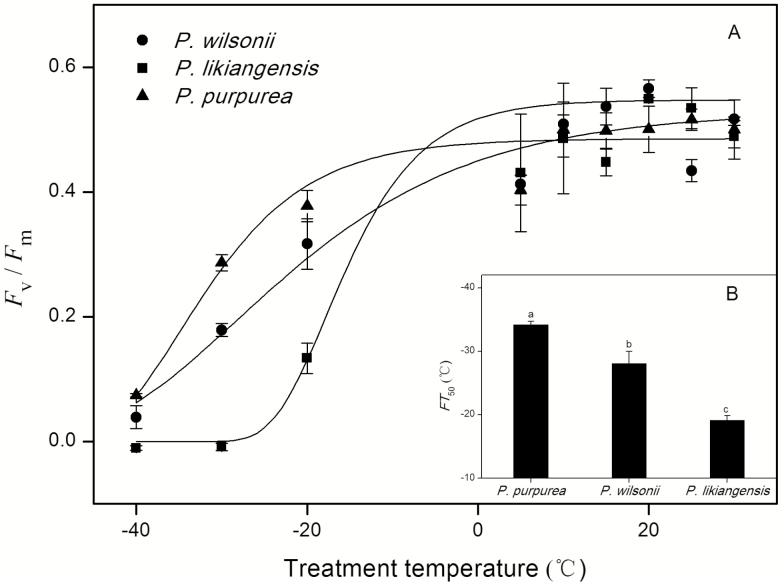
Chlorophyll fluorescence (*F*_v_/*F*_m_) after different temperature treatments for three *Picea* species (A). The temperatures causing a 50 % reduction in initial *F*_v_*/F*_m_ (FT_50_) of three *Picea* species are shown in the inset (B). Significant interspecies differences are indicated with different letters (*P* < 0.05). Data points are presented as means ± SE (*n* = 4).

For all three species, the photochemical efficiency of PSII (*F*_v_/*F*_m_) decreased significantly with the temperature, but the curve for *P. likiangensis* exhibited the steepest downward slope (Fig. 2). Despite this, there were significant inter-species differences in the temperature causing a 50 % reduction in initial *F*_v_/*F*_m_ (FT_50_), with *P. purpurea* having a lower FT_50_ (and thus higher frost tolerance) than its parental species (Fig. 2).

## Discussion

Our results show that the studied species exhibit inherent differences in physiological traits relating to frost tolerance such as the capacity for leaf turgor maintenance at low water potentials and the frost tolerance of the photosynthetic apparatus. These differences contribute to the greater frost tolerance of the homoploid hybrid species *P. purpurea* relative to its progenitors. These findings provide new insights into the role of leaf water relations and leaf frost tolerance in enabling a diploid hybrid species to occupy a novel extreme habitat and promoting the niche separation between the hybrid line and its parents.

As noted in the Introduction, the habitat of the diploid hybrid species *P. purpurea* is characterized by low temperatures, unlike those of its progenitors (Table 1). We found that *P. purpurea* exhibited greater leaf photosystem stability under freezing stress (i.e. it had lower FT_50_ values) than its progenitors (Fig. 2). Similarly, Ma *et al.* (2013) showed that the well-documented diploid hybrid species *P. densata* exhibited greater cold photosynthesis tolerance than its progenitors, suggesting that improvements in the leaves’ photosynthetic apparatus activity in cold climates facilitate the colonization of extreme habitats by homoploid hybrid species. It should be noted that this experiment was done in winter, and frost stress may already reduce the photosystem capacity of needles, which may result in the initial *F*_v_/*F*_m_ values below 0.8. This phenomenon has also been reported in many conifer species, such as red spruce (*Picea rubens*; Lawson *et al.* 2000), black spruce (*Picea mariana*; Gaumont-Guay *et al.* 2003) and coastal Douglas-fir (Rose and Haase 2002).

*Picea purpurea* also exhibited lower *π*_o_ values than its parental species, suggesting an effective osmotic adjustment strategy. Elevated cellular osmotic potentials are associated with adaptation to life in regions where frost stress is common because they reduce the risk of freezing-induced intracellular ice formation, which causes cellular dehydration and cell collapse. This is consistent with previous reports showing that species from regions with limited water availability (including those subject to freezing-induced drought) tend to have more negative *π*_o_ values than species occupying wetter regions (Sack *et al.* 2003; Lenz *et al.* 2006; Bartlett *et al.* 2012). Likewise, the *π*_o_ of *P. likiangensis* was more negative than that of *P. wilsonii*, which may due to the significantly lower precipitation in the driest month for *P. likiangensis* than that for *P. wilsonii* (Table 1).

Maintaining turgor is vital for plant growth, so the more negative a plant’s turgor loss point, the greater its ability to sustain its ecological and physiological activity at low soil and tissue water potentials (Baltzer *et al.* 2008). The lower Ψ_TLP_ value of *P. purpurea* thus confers a survival advantage when colonizing a water-limited habitat. In a recent review, Bartlett *et al.* (2012) proposed three ways by which plants could make their Ψ_TLP_ more negative: reducing *π*_o_, increasing cell wall flexibility (i.e. reducing *ε*) and increasing the extracellular water content. However, we found that only *π*_o_ varied in a manner comparable to that of Ψ_TLP_; the *ε* of *P. purpurea* did not differ significantly from those of its progenitors. *Picea purpurea* also has a comparatively low SWC, meaning that its system for buffering the symplasm against water loss is relatively inefficient. However, this adaptation confers a survival advantage in cold alpine habitats by reducing susceptibility to winter freezing (Beck *et al.* 2007; Baltzer *et al.* 2008). The relatively low SWC of *P. purpurea* may also be counterbalanced by its low stem Ψ_12_, which enables effective xylem hydraulic conductance at strongly negative water potentials.

The upper vulnerability threshold Ψ_12_ is related to the air entry pressure. In the context of stomatal control of transpiration, this variable limits the maximum xylem tension to a level close to but below the threshold for embolism formation under non-extreme conditions in stems with attached leaves (Sparks and Black 1999; Brodribb and Holbrook 2003; Domec *et al.* 2008; Meinzer *et al.* 2009). In alpine and high latitude regions, plants primarily lose water through their leaf surfaces due to the transpiration induced by strong solar radiation, placing them at risk of losing hydraulic conductance as a result of uncontrolled transpiration (Körner 2003). The negatively transgressive stem Ψ_12_ of *P. purpurea* thus indicates strong stomatal regulation of xylem pressure in this species, and hence a larger hydraulic safety margin under non-extreme conditions.

Generally, larger hydraulic safety margins in plants are associated with lower hydraulic conductance, small conduits or tracheid, greater wood densities and more robust wall reinforcement in the xylem (Hacke and Sperry 2001). We found that the stem *K*_max_ of *P. purpurea* was significantly lower than those of its parental species, implying that this would have effects on the twig growth of *P. purpurea*: the lower stem *K*_max_ will limit the water transported from roots to terminal shoots, thus reducing the gas exchange and the capacity of carbon gain of leaves, which further limits the growth of seedlings. Likewise, the high value of twig length in *P. likiangensis* may benefit from its higher stem *K*_max_ (Tables 2 and 3). Moreover, since there is a well-established trade-off between hydraulic efficiency and safety, this suggests that *P. purpurea* tends to exhibit a higher wood density and smaller tracheid than its progenitors, both of which contribute to its larger hydraulic safety margins (Sperry *et al.* 2006; Blackman *et al.* 2010).

**Table 3.** Comparison of P-V and PLC parameters between *Picea purpurea* and its parental species. Significant interspecies differences are indicated with different letters (*P* < 0.05). Data are presented as means ± SE (*n* = 5).

Symbol	*P. purpurea*	*P. wilsonii*	*P. likiangensis*
Leaf P-V parameters			
SWC	1.26 ± 0.02 b	1.46 ± 0.01 a	1.50 ± 0.04 a
Π_o_ (MPa)	−2.05 ± 0.06 c	−1.40 ± 0.10 a	−1.71 ± 0.10 b
Ψ_TLP_ (MPa)	−2.68 ± 0.09 b	−2.12 ± 0.04 a	−2.26 ± 0.12 a
RWC_TLP_ (%)	85.97 ± 1.21 a	87.66 ± 0.55 a	87.69 ± 1.75 a
*ε* (MPa)	15.29 ± 0.61 a	12.59 ± 0.97 a	14.41 ± 1.27 a
*C*_FT_ (MPa)	0.05 ± 0.002 a	0.05 ± 0.002 a	0.05 ± 0.005 a
PLC parameters			
Leaf Ψ_12_ (MPa)	−1.06 ± 0.25 a	−1.05 ± 0.15 a	−0.79 ± 0.18 a
Leaf Ψ_50_ (MPa)	−2.12 ± 0.15 b	−2.04 ± 0.19 b	−1.31 ± 0.19 a
Leaf Ψ_88_ (MPa)	−3.18 ± 0.27 b	−3.03 ± 0.24 b	−1.82 ± 0.26 a
Leaf *K*_max_ (mmol MPa^−1^ s^−1^ m^−2^)	1.28 ± 0.24 a	1.03 ± 0.25 a	1.33 ± 0.30 a
Leaf Ψ_mid_ (MPa)	−0.93 ± 0.02 a	−0.99 ± 0.04 a	−0.83 ± 0.08 a
Stem Ψ_12_ (MPa)	−2.12 ± 0.29 c	−1.38 ± 0.15 b	−0.26 ± 0.17 a
Stem Ψ_50_ (MPa)	−2.78 ± 0.1 b	−2.79 ± 0.1 b	−1.77 ± 0.13 a
Stem Ψ_88_ (MPa)	−3.46 ± 0.35 a	−4.02 ± 0.09 a	−3.27 ± 0.08 a
Stem *K*_max_ (mol MPa^−1^ s^−1^ m^−1^)	8.21 ± 0.12 c	15.51 ± 0.64 b	27.23 ± 1.08 a
Leaf Ψ_50_ − Stem Ψ_50_ (MPa)	0.67 ± 0.15 a	0.66 ± 0.19 a	0.45 ± 0.19 a
Leaf Ψ_mid_ − Leaf Ψ_50_ (MPa)	1.19 ± 0.02 a	1.05 ± 0.05 a	0.48 ± 0.09 b

Growing evidence from studies comparing hydraulic safety margins in leaves and branches suggests that species with branches that rely more heavily on structural avoidance of embolism tend to have leaves that lose hydraulic function on a daily basis (Woodruff *et al.* 2007; Johnson *et al.* 2009, 2011). In this study, there were clear differences between the three *Picea* species with respect to the vulnerability of their leaves and stems to losses of hydraulic function, and the leaves were generally more vulnerable than the stems (Fig. 1A and B). This is consistent with the hypothesis that embolism resistance in the distal segments is reduced to maximize the safety of the more proximal stems (Johnson *et al.* 2012). Leaf Ψ_50_ has been linked to plant survival, and stem Ψ_50_ has been shown to be an adaptive trait across broad taxonomic groups in relation to gradients in water availability (Blackman *et al.* 2009; Brodribb and Cochard 2009). Therefore, the comparatively negative leaf Ψ_50_ and stem Ψ_50_ values of *P. purpurea* and *P. wilsonii* suggest adaptation to habitats with variable water availability. However, *P. wilsonii* prefers warmer habitats, whereas *P. purpurea* colonizes colder ones. Prolonged droughts can occur during summer because elevated temperatures increase transpiration at leaf surfaces, which could cause *P. wilsonii* to suffer from drought stress, whereas *P. purpurea* is frequently exposed to frost drought in winter. It has been known for many years that drought conditions can induce cold hardiness in plants, and that exposure to cold may improve drought resistance (Thomas and James 1993). This may explain the similarity of *P. purpurea* and *P. wilsonii* with respect to their leaf Ψ_50_ and stem Ψ_50_ values (Table 3). In contrast, the Ψ_TLP_ and *π*_o_ between these two species were significantly different. We suggest the following explanation: because the leaf hydraulic vulnerability to water stress is controlled by both xylem and outside-xylem, the leaf Ψ_50_ is typically less negative than the turgor loss point (Sack *et al.* 2016; Scoffoni *et al.* 2017). *Picea purpurea* has a higher LMA (data unpublished), indicating its tissue solute was relatively high and the resistance of water transport through outside-xylem would be larger than that of *P. wilsonii*. However, the needles of twigs of *P. purpurea* are relatively small and dense; thus, the distance between xylem and water transpiration sites (stomata) would be shorter. This would lead to the leaf and stem hydraulic conductance (the leaf Ψ_50_ and stem Ψ_50_) of *P. purpurea* not differing significantly from that of *P. wilsonii*, but their *π*_o_ and Ψ_TLP_ were significantly different.

Over the course of a day, the xylem pressure experienced by the leaves is generally lowest at midday. The differences between Ψ_mid_ and the leaf Ψ_50_ in *P. purpurea* and *P. wilsonii* were also greater than in *P. likiangensis*, also suggesting that the former two species have larger hydraulic safety margins (Table 3). The stem Ψ_50_ values used in the safety margin calculations were the mean values for the studied species, which may explain why the magnitude of the difference between the leaf Ψ_50_ and stem Ψ_50_ values was so similar for all three species (Table 3). It should be noticed that the curves plotted in Fig. 1 were re-fitted using all of the data for each species, instead of using a simple duplicate of individuals. That is why the values in the curves were slightly different from Table 3. Besides, although the initial points of three *Picea* species were at the origin (0 MPa, 0 % PLC), the stem PLC curves of *P. likiangensis* were not at the origin (Fig. 1B). This may be because the stem PLC of *P. purpurea* and *P. wilsonii* were well fitted through a sigmoid function, while for *P. likiangensis*, the stem PLC was much closer to a logarithmic function.

The greater ability to withstand cellular desiccation and superior frost tolerance in leaf photosynthetic apparatus in *P. purpurea* may be the result of transgressive segregation during the hybridization process, which later became fixed as populations bearing these traits stabilized as a homoploid hybrid species. Molecular evidence indicates that *P. purpurea* originated at around the same time as the initiation of the largest Quaternary glaciation on the QTP, and that it subsequently underwent an extensive population expansion on the QTP (Sun *et al.* 2014). During this period, *P. wilsonii* and *P. likiangensis* may have returned to their former geographical ranges, allowing the new hybrid linage *P. purpurea* to occupy areas at higher altitudes and latitudes than its progenitors (Sun *et al.* 2014). Therefore, it is reasonable to conclude that these cold-tolerant traits promoted the initial ecological separation of *P. purpurea* from its parental species and then facilitated its extensive population expansion, and that the latter process may have been reinforced during the period of glaciation.

## Conclusions

In this study, we examined the differences in needle frost tolerance of photosystem stability (*F*_v_/*F*_m_), water relations and xylem resistance to dysfunction (both for leaves and stems) between the homoploid hybrid species *P. purpurea* and its parental species. The results showed that *P. purpurea* has higher leaf photosynthetic stability frost tolerance and greater capacity to withstand cellular desiccation than its parental species. These traits may have contributed to its adaptation to higher altitude and latitude regions where frost stress is frequent. We also noticed that the xylem resistances of leaves and stems of *P. purpurea* were similar to those of one of its parental species *P. wilsonii* and larger than those of another. This phenomenon may relate to their specific habitat: although *P. wilsonii* prefers warmer habitats and *P. purpurea* colonizes colder ones, both of them would experience water stress (induced by drought and cold, respectively).

## Sources of Funding

This study was funded by the National Natural Science Foundation of China (31170571, 31370603 and 31522013) and the Fundamental Research Funds for the Central Universities (lzujbky-2016-ct10).

## Contributions by the Authors

J.W., M.W. and X.Z. designed this research; J.W., M.W. and X.Z. performed the experiment and analyzed the data; J.W. wrote the manuscript; S.S. and C.Z. revised the manuscript.

## Conflicts of Interest Statement

No conflicts of interest.
